# Organelle Genome Inheritance in *Deparia* Ferns (Athyriaceae, Aspleniineae, Polypodiales)

**DOI:** 10.3389/fpls.2018.00486

**Published:** 2018-04-13

**Authors:** Li-Yaung Kuo, Te-Yen Tang, Fay-Wei Li, Huei-Jiun Su, Wen-Liang Chiou, Yao-Moan Huang, Chun-Neng Wang

**Affiliations:** ^1^Institute of Ecology and Evolutionary Biology, National Taiwan University, Taipei, Taiwan; ^2^Taiwan Forestry Research Institute, Taipei, Taiwan; ^3^Boyce Thompson Institute, Ithaca, NY, United States; ^4^Plant Biology Section, Cornell University, Ithaca, NY, United States; ^5^Department of Earth and Life Sciences, University of Taipei, Taipei, Taiwan; ^6^Dr. Cecilia Koo Botanic Conservation Center, Pingtung, Taiwan; ^7^Department of Life Science, National Taiwan University, Taipei, Taiwan

**Keywords:** *Deparia*, eupolypod, fern, maternal inheritance, mitogenome, plastome, tissue-direct PCR

## Abstract

Organelle genomes of land plants are predominately inherited maternally but in some cases can also be transmitted paternally or biparentally. Compared to seed plants (>83% genera of angiosperms and >12% genera of gymnosperms), plastid genome (plastome) inheritance has only been investigated in fewer than 2% of fern genera, and mitochondrial genome (mitogenome) from only one fern genus. We developed a new and efficient method to examine plastome and mitogenome inheritance in a fern species—*Deparia lancea* (Athyriaceae, Aspleniineae, Polypodiales), and found that plastid and mitochondrial DNAs were transmitted from only the maternal parentage to a next generation. To further examine whether both organelle genomes have the same manner of inheritance in other *Deparia* ferns, we sequenced both plastid and mitochondrial DNA regions of inter-species hybrids, and performed phylogenetic analyses to identify the origins of organellar DNA. Evidence from our experiments and phylogenetic analyses support that both organelle genomes in *Deparia* are uniparentally and maternally inherited. Most importantly, our study provides the first report of mitogenome inheritance in eupolypod ferns, and the second one among all ferns.

## Introduction

Unlike the strict maternal inheritance of mitochondrial genomes in animals, organelle inheritance in land plants are complex and variable. Plant plastid and mitochondrial genomes (plastome and mitogenome, respectively) can be inherited maternally but also paternally or biparentally. In angiosperms, maternal inheritance is believed to be predominant in both organelle genomes; however, in around 20% of genera, plastomes were found to be putatively biparentally inherited (Mogensen, 1996; Zhang and Sodmergen, 2010; Jansen and Ruhlman, 2012; Choubey and Rajam, 2015). In a few angiosperm taxa, cases of biparental inheritance of the mitogenome and paternal inheritance of the plastome were confirmed (Zhang and Sodmergen, 2010; Li et al., 2013; Mccauley, 2013; Choubey and Rajam, 2015). In gymnosperms, both organelles are maternally inherited in non-conifer lineages, including cycads, *Ginkgo*, and gnetophytes (Mogensen, 1996; Jansen and Ruhlman, 2012). In contrast, for some conifers, paternal and biparental inheritance of the plastome, and paternal inheritance of the mitogenome was identified (Mogensen, 1996; Jansen and Ruhlman, 2012; Worth et al., 2014).

In seed-free land plants, such as ferns and bryophytes, organelle genomes have been found to be only maternally inherited (reviewed in Zhang and Sodmergen, 2010). However, the variability of organelle genome inheritance for these plants is likely to be underestimated due to a poor and disproportional sampling. In ferns, while maternal inheritance is generally assumed, genetic evidence is weak—only four cases were studied for fern plastome inheritance, and only one was for that of mitogenome (Gastony and Yatskievych, 1992; Vogel et al., 1997; Guillon and Raquin, 2000; Adjie et al., 2007). The proportion of examined fern genera is less than 2% (*sensu* PPG, 2016), and is far smaller than that in either angiosperms or gymnosperms (respectively estimated to be >83% and >12% of genera; Reboud and Zeyl, 1994; Mogensen, 1996; Zhang et al., 2003; Worth et al., 2014).

Cytologically, maternal inheritance of the organelle genomes in some ferns is implicated by the anatomical ontology during fertilization. Both plastids and mitochondria exist in fern egg cells and functional sperms (Duckett, 1973; Raghavan, 1989 and references listed therein; Kotenko, 1990; Gori et al., 1997; Muccifora et al., 2000; Renzaglia et al., 2001; Lopez-Smith and Renzaglia, 2002, 2008; Sakaushi et al., 2003; Cao et al., 2009, 2010; Wolniak et al., 2011; Cao, 2014), but plastids from sperm are known to be excluded before immersion into an egg (Bell and Duckett, 1976; Lopez-Smith and Renzaglia, 2002, 2008; Cao et al., 2010, 2016). In *Osmunda, Pteridium*, and *Ceratopteris*, mitochondria from sperms are digested soon after fertilization (Bell and Duckett, 1976; Lopez-Smith and Renzaglia, 2008; Cao et al., 2010, 2016). However, in *Lygodium*, paternal mitochondria are still retained after fertilization, and it is unclear whether these mitochondria persist in subsequent developmental stages (Lopez-Smith and Renzaglia, 2002).

**FIGURE 1:**
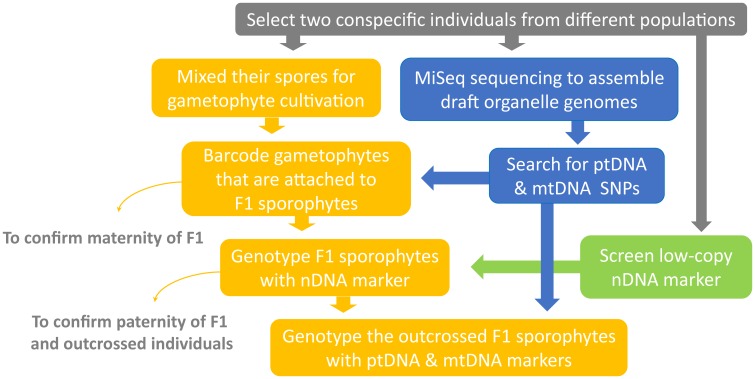
Experimental workflow of the current study.

**FIGURE 2:**
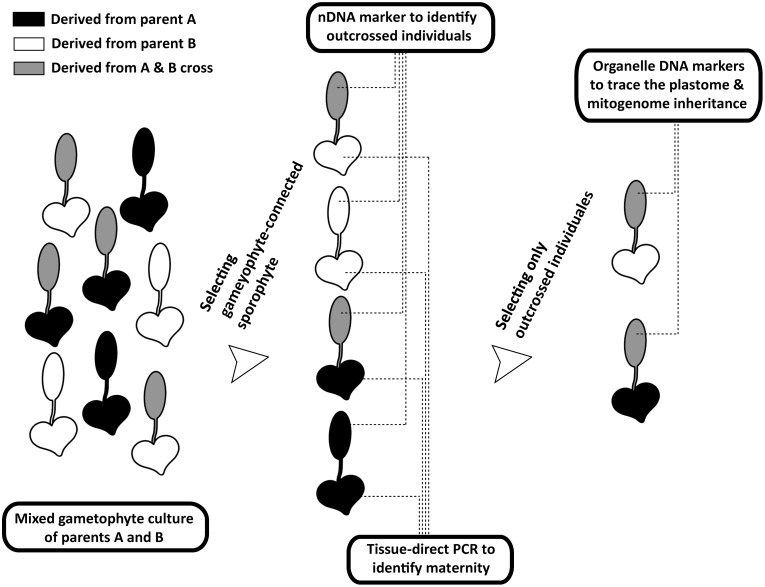
Procedures of tracing organelle genome transmission in the current study. The heart- and spoon-shaped icons respectively indicate gametophyte and sporophyte juveniles of *Deparia lancea*. The white and black ones respectively indicate descendants from parent A or B, while the gray ones indicate outcrossed sporophytes between parents A and B.

This study aims to broaden the understanding of organelle genome inheritance in ferns by exploring an additional and uninvestigated lineage—*Deparia* (Athyriaceae, Aspleniineae, Polypodiales). Most importantly, we provide the second confirmed case of maternal inheritance of mitogenome in ferns, and the first one for that in the eupolypods, the most species-rich lineage in ferns (PPG, 2016). In addition, we developed a new genetic experiment (**Figures 1, 2**) that can greatly facilitate the investigations on organelle inheritance in seed-free plants like ferns, which mostly produce small and hermaphroditic gametophytes, and therefore, are difficult to be manipulated in outcrossing experiments to trace organelle genome transmissions. Given that ferns belong to the extant lineage most closely related to seed plants, an improved understanding of ferns should provide valuable insights into the evolution of variable organelle genome inheritance in land plants.

## Materials and Methods

### Overview of Experimental Design

We developed a new genetic-based method to trace organelle genome transmission in ferns (**Figures 1, 2**). First, we selected two conspecific and sexual individuals (given names of “A” and “B” in **Figure 2**) from different populations as our parental sources. Next, we mixed their spores, and cultivated their gametophytes together until F1 sporophyte offspring were generated. At the same time, we applied a genome skimming approach (**Figure 1** blue part) to seek single-nucleotide polymorphisms (SNPs) among their plastomes and mitogenomes, and found a plastid (pt)DNA marker and a mitochondrial (mt)DNA marker that can distinguish the two parental individuals. We also screened some candidate nuclear (n)DNA loci to find a diagnostic region as a biparentally inherited nDNA marker. For every individual of F1 sporophyte progenies, we then used these genetic markers to determine (i) its maternity by barcoding its attached gametophyte (i.e., the donor of egg) and (ii) its paternity by the nDNA marker genotyping (**Figures 1, 2**). Since we confirmed both parentages for these individuals, we could identify the outcrossed ones (i.e., crossing between gametophytes from the different parental individuals; **Figure 2**), and select them for further ptDNA and mtDNA genotyping. Compared with that of the two parental individuals, these genotyping results could further infer the parentage(s) that transmitted these organelle genomes from a gametophyte generation to a next sporophyte generation.

### Sample Preparation

Two diploid individuals of *Deparia lancea* from two localities in Taiwan were selected as parental sources: *Kuo4046* from Taichung City and *Kuo4294* from Taoyuan City, which are referred to as parents A and B, respectively. Their ploidies were confirmed by flow cytometry following Kuo (2015). Spores of the two parents were collected from living materials cultivated in a greenhouse of the Taiwan Forestry Research Institute. Their fertile leaves were wrapped in weighing paper, and dried at room temperature for 2–3 days to allow spore release. The collected spores were subsequently stored in 0.5 mL microcentrifuge tubes under 4°C and used within 2 months.

### Organelle Genome Assembly

DNA of both parents were extracted from their leaves by a modified CTAB procedure (Kuo, 2015), and then fragmented into an averages size of 500 bp using Covaris S2 (Covaris, Woburn, MA, United States). We constructed the Illumina libraries using NEBNext DNA Library Prep Master Mix Set (New England Biolabs, Ipswich, MA, United States), which were sequenced on Illumina MiSeq (2 × 300 bp paired-end) producing 0.4–0.6 Gb per sample. We removed the adapter sequences using Scythe (Buffalo, 2014) and trimmed reads to remove low quality bases by Sickle (Joshi and Fass, 2011). To assemble the organelle genomes, we input the reads into NOVOPlasty (Dierckxsens et al., 2017). For the plastome, we used the *Woodwardia* assembly (GenBank accession: NC_028543) as the seed. For the mitogenome, we used the coding exon sequences from *Salvinia* mitogenome (Li et al., unpublished data) as the seeds, and used the *Deparia* plastome assemblies to exclude plastome reads (by “Chloroplast sequence = ” setting). The assembled organelle contigs were validated by read-mapping using bwa (Li and Durbin, 2009), and annotated in Geneious (Kearse et al., 2012). These raw reads were deposited in NCBI Short Read Archive (SRP136489) and the NCBI accessions for organelle contigs are: MH124207-35.

### SNP Identification

To identify SNPs in organelle genomes between the two parental individuals, we re-mapped their Illumina reads to our plastome and mitogenome assemblies using bwa (Li and Durbin, 2009), and then inspected the mapping results in Geneious. We found SNPs in the plastid *ndh*F (NADH-plastoquinone oxidoreductase subunit five) and mitochondrial *nad*9 (NADH dehydrogenase subunit nine) genes (Supplementary Figures S1, S2), which were then respectively selected as the ptDNA marker and the mtDNA marker. Primers were designed to target these two regions (**Table 1**).To find a nDNA marker, we screened the low-copy loci from Rothfels et al. (2013), and found a diagnostic SNP at the 13th intron of *IBR3* (IBA-response 3) (Supplementary Figure S3). To verify the sequences of the two *IBR3* alleles in each parental individual, we used HiFi DNA polymerase (Kapa Biosystems, Wilmington, DE, United States) for PCR amplification, and cloned the PCR products into the pJET1.2/blunt cloning vector (Thermo Fisher Scientific, Waltham, MA, United States). Ligation, transformation, plating, and selection of clones followed the manufacturer’s protocol. All primer information is summarized in **Table 1**. All the generated sequences were deposited in GenBank (Accession Nos.: MG972633-40).

**Table 1:** Primer information.

Primer	Target taxon	Genetic Region^a^	5′-3′ sequence	Reference
Dl IBR3 fCSI	*Deparia lancea*^b^	*IBR*3	CAACAAACATTTCCTGCTCAATCAG	This study
Dl IBR3 rPGR	*Deparia lancea*^b^	*IBR*3	CAATGGTGGAGTCTTCCTGG	This study
AT IBR fPDV	Athyriaceae^c^	*IBR*3	GCAATGACTGAACCAGATGTG	This study
AT IBR rAER	Athyriaceae^c^	*IBR*3	ATSTCTATCCCACGCTCAGC	This study
De ndhF fCGK	*Deparia lancea*^b^	*ndh*F	GGGGACTTAATTTGTGGAAAGG	This study
Del ndhF rPSL	*Deparia*^c^	*ndh*F	CCATAAGGGATAAACTAAGCGAAG	This study
Del nad9 fFAI	*Deparia lancea*^b^	*nad*9	ATGACTTGCAGTCCACTTGAATAATTTGCTATTG	This study
Del ndh9 rPWR	*Deparia lancea*^b^	*nad*9	GGACGGCATTAGTCGCCAAGG	This study
FernLr1	*Deparia*^c^	*trn*L-L-F	GGCAGCCCCCAGATTCAGGGGAACC	Li et al., 2011
f	Plants^c^	*trn*L-L-F	ATTTGAACTGGTGACACGAG	Taberlet et al., 1991
FERpl2 fTFF	Ferns^c^	*rpl*2 intron	CACCTTTTTCCGATGTCAC	This study
De rpl2 rGGD	*Deparia*^c^	*rpl*2 intron	GGCGTAGTCTCCTCCAG	This study


*^a^IBR3, IBA-response 3; ndhF, NADH-plastoquinone oxidoreductase subunit five; nad9, NADH dehydrogenase subunit nine; trnL-L-F, tRNA-Leu intron + tRNA-Leu-to-tRNA-Phe intergenic spacer; rpl2, ribosomal protein L2. ^b^Specific primer only for Deparia lancea. ^c^Universal primer.*

### Culture for Gametophyte and Sporophyte Progeny

Spores from both parental individuals were mixed and sowed on the top of soil medium in a 7.5 × 9-cm plastic box (PHYTATRAY II^TM^ no. P5929; Sigma, St. Louis, MO, United States) with a density of roughly 320∼350 spores/cm^2^. The soil medium contained a mixture of vermiculite: peat: perlite in a 2:2:1 volume ratio. After 3 months when the number of sporophyte offspring seemed saturated and no newly generated sporophyte individual was found, we transferred the gametophyte-connected sporophytes into individual plots. Both gametophytes and sporophytes were cultured under LED white fluorescent illumination of 6.3 ± 0.3 μmole m^-2^ s^-1^ for 10 h d^-1^, and the daily temperature ranged 20∼28°C. The humidity was monitored to avoid desiccation of the cultures.

### Confirming Sexual Reproduction

We first checked the spore number per sporangium (S/S) of both parental individuals to infer their reproductive modes. In *Deparia*, 64 S/S and 32 S/S are respectively indicative of sexual and apomictic individuals (Kato et al., 1992). In addition, we conducted flow cytometric analyses of both the gametophyte and sporophyte offspring to confirm their relative nuclear genome size, as well as their reproductive mode (i.e., with sexual reproduction, gametophytes should have a genome size half that of sporophyte progeny). Twenty gametophyte individuals (each around 0.5 cm^2^ in size) without a juvenile sporophyte were used for the flow cytometric analysis to confirm the gametophyte genome size; while leaf tissues of sporophyte juveniles were used to confirm the sporophyte genome size. The flow cytometric method followed Kuo (2015).

### Determining Maternity of F1 Sporophyte Progeny

To determine whether the gametophyte-attached sporophyte progeny was derived from parent A or B, we used the partial *ndh*F as the DNA marker and a PCR-RFLP (restriction fragment length polymorphism) approach for identification. The *ndh*F products of these gametophytes were first amplified using tissue-direct PCR following Li et al. (2010). Then, 1 or 2 μL from each of these PCR products was treated with 5 U of the restriction enzyme of AciI (New England Biolabs, Ipswich, MA, United States) at 37°C for 60 min and then 65°C for 20 min. The AciI-treated *ndh*F products were subsequently examined by electrophoresis using 1× TBE and a 1.5% agarose gel at 110 V for 45 min. After electrophoresis, the gel was then stained in an ethidium bromide solution for 10 min. The gametophytes from parent A would have two DNA fragments on an electrophoresis gel (at 130 and 156 bp; Supplementary Figure S1), while those from parent B would have a single undigested 286 bp band.

### Identification of Sporophyte Progeny Resulting From Outcrossing

After the sporophyte offspring became mature and they were large enough to produce spores, their genomic DNAs were extracted, using a modified CTAB procedure following Kuo (2015). We amplified the *IBR*3 sequences from these extracted DNAs. We performed a PCR in 15-μL volume reactions, including 20 ng of genomic DNA, 1 × PCR buffer 200 μM dNTP, 15 pmol of each primer, and 1 U polymerase (ExPrime Taq DNA Polymerase; GENETBIO, Daejeon, Korea). Because there is one SNP in the 13th intron that can distinguish between parents A and B (at position 248 in Figure S3), we directly sequenced these *IBR3* amplicons, and used the presence of a double-peak signal to identify sporophyte individuals resulting from inter-gametophytic outcrossing.

### Tracing Organelle Genome Inheritance Using ptDNA and mtDNA Markers

Genotyping of the ptDNA and mtDNA markers was conducted only on those outcrossed sporophyte offspring, and the results were compared to those of the parents to confirm their organelle genome inheritance. The partial *ndh*F and the partial *nad*9 (respectively as the ptDNA and mtDNA markers) were amplified from genomic DNAs of these sporophyte offspring. The PCRs were conducted as described in the previous section. A single band from *ndh*F PCR-RFLP indicates that ptDNA was derived from parent B (see the detailed method in “Determining Maternity of F1 Sporophyte Progeny”). On the other hand, when DNA fragments with the sizes of 130 and 156 bp were found, we additionally sequenced these *ndh*F products to discern that if they contained sequences from only parent A or from both parents. The *nad*9 genotypes of outcrossed sporophyte offspring were determined by sequencing.

### Organelle Genome Inheritance in Other *Deparia* Ferns

To reveal possible manners of organelle genome inheritance in other *Deparia* ferns, we reconstructed both ptDNA and mtDNA phylogenies for some hybrids between the AT and DE clades (i.e., *D*. ×*tomitaroana*, *D.* ×*nakaikeana*, and *D.* × *lobatocreneta*; Kuo et al., 2018). By identifying the phylogenetic origins of their ptDNA and mtDNA, we can confirm whether these DNAs came from one or both parentages during hybridizations. *trn*L-L-F (including *trn*L gene and *trn*L-F intergenic spacer) and *rpl*2 intron were selected to reconstruct their ptDNA and mtDNA phylogenies, respectively. In total, ten *D*. ×*tomitaroana*, one *D.* × *nakaikeana*, one *D.* ×*lobatocreneta* individual, and 11 additional *Deparia* species (three from DE clade, four from AT clade, and one representative from each of the other clades; *sensu* Kuo et al., 2018) were sampled. *Woodwardia radican*s and *Oceaniopteris gibba* from Blechnaceae were selected as outgroups. We followed Li et al. (2011) for amplification and sequencing of *trn*L-L-F, and for *rpl*2 intron new primers were designed (**Table 1**). The voucher information and GenBank accessions for these samples are in Supplementary Table S1. The maximum likelihood phylogenies of these genes were reconstructed using IQ-TREE web server (Trifinopoulos et al., 2016) with 1000 ultrafast bootstrap replicates.

## Results

### Confirmation of Sexual Reproduction and Random Mating

**FIGURE 3:**
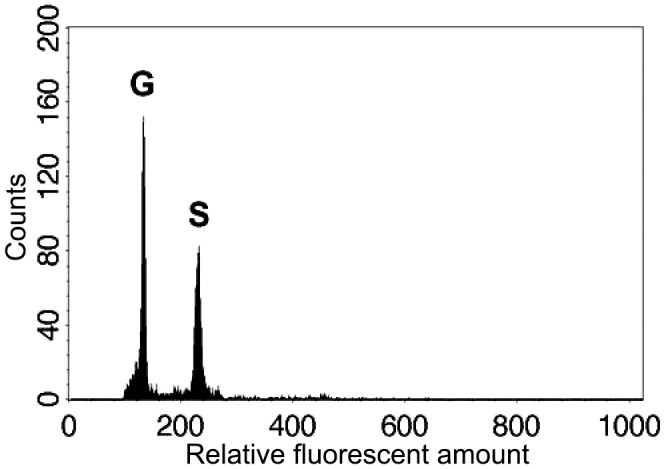
Relative genome sizes of the gametophyte (G) and sporophyte (S) of the diploid *Deparia lancea* examined by flow cytometry.

In both parental individuals of *Deparia lancea*, we found that only 64-spored sporangia were developed. Like other *Deparia* ferns (Kato et al., 1992), 64-spored sporangia indicate sexual reproduction, and produce spores that are half the ploidy level. Flow cytometric results further confirmed that these gametophytes had half the genome size compared to their sporophyte offspring (**Figure 3**). No indication of apomixis was found in the sampled individuals.

In total, we genotyped 65 pairs of gametophyte and F1 sporophyte offspring. Overall, the resulting sporophyte offspring displayed a pattern of random mating, in which the observed numbers of outcrossed and selfed individuals (**Table 2**) do not significantly deviate from the expected numbers (Chi-squared *p* > 0.90). Among the outcrossed individuals, 13 had A as the maternal parent and 11 had B (**Table 2**); and this result revealed no significant bias in maternal parentage (Chi-squared *p* > 0.60), which further suggests that neither asymmetric mating nor cytonuclear incompatibility likely occurred among infraspecific and homoploidy crossings in *Deparia lancea.*

**Table 2:** Genotyping results of sporophyte F1 offspring.

F1 offspring^a^	Maternal parent^b^	Offspring ptDNA	Offspring mtDNA	Number of individuals
Outcrossed	A	Parent A^b^	Parent A^c^	13
Outcrossed	B	Parent B^b,c^	Parent B^c^	11
A selfed	A	–	–	3
B selfed	B	–	–	38

*^*a*^Confirmed by *IBR3* sequences. ^*b*^Confirmed by *ndh*F PCR-RFLP. ^*c*^Confirmed by sequencing.*

### Identification of Outcrossed Progeny and ptDNA and mtDNA Genotype

The numbers of outcrossed and inbred progenies are summarized in **Table 2**. In total, 37% of the sporophyte offspring were identified as outcrossing between parents A and B. Among the outcrossed offspring, 54 and 46% individuals had a maternal parent of A and of B, respectively. All their ptDNA and mtDNA genotypes are same as those of their maternal parents.

### ptDNA and mtDNA Phylogenies

**FIGURE 4:**
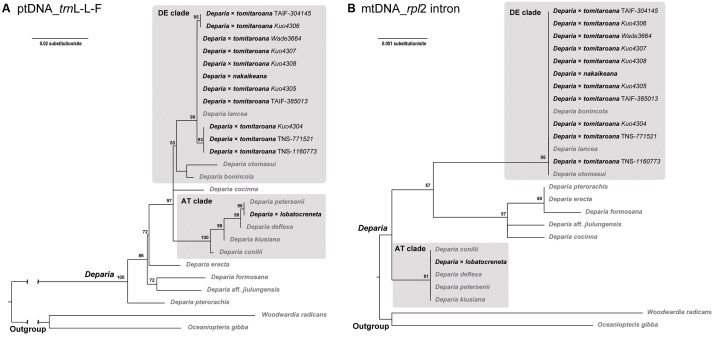
**(A)** ptDNA (*trn*L-L-F) and **(B)** mtDNA (*rpl*2 intron) maximum likelihood phylogenies of the AT × DE inter-clade hybrids in *Deparia*. The values on branches are their bootstrap supports, and only the values larger than 50 are shown. The inter-clade hybrids are indicated with black names, and the other taxa are indicated with gray names.

The alignment of plastid *trn*L-L-F contained 889 characters with 25% of variable sites, and that of mitochondrial *rpl*2 intron contained 1384 characters with 1.4% of variable sites. The phylogenies inferred from these two regions are shown in **Figure 4**. In both phylogenies, all *D.* ×*tomitaroana* individuals and *D.* × *nakaikeana* are nested in the DE clade. For *D.* ×*lobatocreneta*, it is nested in the AT clade. In every of these hybrid individuals, we found ptDNA and mtDNA were inherited uniparentally from a same clade. In other words, ptDNA and mtDNA have the same manner of inheritance in these *Deparia* ferns.

## Discussion

### Advantages and Limitations of the Current Approach

To directly infer organelle genome inheritance in land plants, artificial crosses and subsequent verification of the genomic constitution in the progenies have been commonly used. However, carrying out directional crosses with specific maternity and paternity is very difficult in ferns. Unlike seed plants, ferns are mostly homosporous (Haufler et al., 2016) and we cannot easily manipulate the gametophyte sex to ensure that sperms are coming from one gametophyte to the other. Moreover, it is almost impossible to infer organelle genome transmission in ferns by examining the organelle identity of germ line cells (e.g., eggs or sperms). Generally, mitochondria and plastids are present in both their sperms (or spermatocytes) and eggs (Duckett, 1973; Raghavan, 1989 and references therein; Kotenko, 1990; Gori et al., 1997; Muccifora et al., 2000; Renzaglia et al., 2001; Lopez-Smith and Renzaglia, 2002, 2008; Sakaushi et al., 2003; Cao et al., 2009, 2010; Wolniak et al., 2011; Cao, 2014 and references therein), thus any manner of organelle inheritance—maternal, paternal, and even biparental—is possible.

To overcome the difficulty of ascertaining maternity and paternity involved in a cross in fern species, two approaches were previously adopted. One common approach is to manipulate female and male gametophyte pairings for outcrossing (i.e., a hybrid between gametophyte individuals from different parents). In practice, such experiments need to first confirm the sex of the gametophyte individuals based on a gametangium observation, then separately select female and male individuals from sex-mixed gametophyte cultures of identified sources, and pair them for outcrossing (e.g., Lovis, 1968; Guillon and Raquin, 2000; Yatabe et al., 2001). The genetic compositions of these resulting hybrids are further confirmed by biparentally inherited genetic markers (i.e., nuclear DNA markers) to exclude inbred progeny from outcrossed ones, because cultured female or male gametophytes might subsequently become bisexual. Such a method was used to trace plastome inheritance in several previous studies (Vogel et al., 1997; Guillon and Raquin, 2000; Adjie et al., 2007; **Table 3**). In the other approach (Gastony and Yatskievych, 1992), the paternal parentage of a fern hybrid species was identified as the sperm donor because this parental species was unable to produce functional eggs due to apomixis (reviewed in Gastony and Windham, 1989). The inheritance of organelle genomes in these hybrids were then revealed by ptDNA and mtDNA markers. However, these approaches mentioned above are labor-intensive for manipulating pairings of gametophyte individuals, or, for the latter case, have additional limitations requiring a cross between apomictic and sexual taxa.

**Table 3:** Organelle genome inheritance in ferns.

Genus (family)	Order/suborder	Organelle genome	Inheritance	Reference
*Equisetum* (Equisetaceae)	Equisetales	Plastome	Maternal	Guillon and Raquin, 2000
*Ceratopteris* (Pteridaceae)	Polypodiales/Pteridineae	Plastome	Maternal	Adjie et al., 2007
*Pellaea* (Pteridaceae)	Polypodiales/Pteridineae	Plastome and mitogenome	Maternal	Gastony and Yatskievych, 1992
*Asplenium* (Aspleniaceae)	Polypodiales/Aspleniineae	Plastome	Maternal	Vogel et al., 1997
*Deparia* (Athyriaceae)	Polypodiales/Aspleniineae	Plastome and mitogenome	Maternal	This study

Here, we develop a new approach which can efficiently confirm the maternity and paternity of an artificial cross/hybrid in ferns while avoiding manual pairing of gametophytes for outcrossing, and can be applied to most ferns and other seed-free plants as well. Because a sporophyte is initiated from a zygote inside an archegonium that is attached to its maternal gametophyte, we can genotype a gametophyte and the attached sporophyte in order to trace the maternal and paternal parentages (**Figure 2**). In this study, we determined the maternity of each sporophyte by barcoding its connected gametophytes using a genetic marker (i.e., *ndh*F PCR-RFLP). We then used nDNA marker (i.e., the SNP at 13th intron of *IBR3*) to identify the paternity. One key feature of our approach was the incorporation of tissue-direct PCR, which requires only a >1 mm^2^ piece of tissue for a single PCR reaction (Li et al., 2010). This methodology enabled us to accomplish the DNA-based identification for gametophytes, thus verifying the maternal donor of sporophyte offspring. In addition, we adopted a genome skimming strategy to search SNPs throughout organelle genomes. Such strategy is more efficient, and better guarantees finding diagnostic regions for ptDNA and mtDNA markers. Especially for mtDNA, because of the limited genetic and genomic information for ferns (Guo et al., 2017) and slow substitution rate in plant (Gualberto and Newton, 2017), a strategy directly designing primers and seeking variable regions is usually not cost-effective.

Like other genetic-based approaches, this current approach requires the generation of outcrossed/hybridized F1 offspring from parental sources with preexisting genetic variation. Therefore, this approach is not applicable for plant species having no or very limited genetic variation at the population level. Another limitation of the current approach is that maternity identification of sporophyte offspring relies on DNA-barcoding the gametophyte which is attached on the sporophyte, and, as a result, female or bisexual gametophytes (i.e., egg donors) must generate with enough tissue amount for a barcoding experiment. Such requirement is very hard to be satisfied for the female gametophytes of heterosporous ferns and lycophytes because these gametophytes are usually endosporous, minute, and composed of a limited number of cells (Raghavan, 1989). Fortunately, because these seed-free plants are heterosporous, their outcrossing can be easily manipulated by coculturing microspores (i.e., only developing into male gametophytes) and megaspores (i.e., only developing into female gametophytes) from two different individuals—a similar way as done for that of the seed plants.

### Organelle Genomes Inheritance in *Deparia* and Other Ferns

In this study, we confirmed that plastome and mitogenome are inherited maternally in *Deparia lancea* (**Table 2**). After sequencing the ptDNA in nearly a half of the outcrossed individuals and mtDNA genotypes in all outcrossed individuals, we found no signal indicating paternal transmission of these genomes (**Table 2**). Based on our mtDNA and ptDNA phylogenies, we further confirmed in the inter-clade *Deparia* hybrids that their plastome and mitogenome inheritance are both uniparental (**Figure 4**), and are most likely maternal. The current study is the second to report mitogenome inheritance in ferns, and the first one in the most diversified fern lineage—the euploypods (Aspleniineae + Polypodiineae). For Aspleniineae plastome, our result is second to Vogel et al. (1997) to support maternal inheritance. In addition, our findings support one of the most important assumptions in fern phylogeny and genetics that ptDNA and mtDNA can only trace the maternal lineage.

Despite the scant case studies, our results show the consistency of maternal inheritance of the organelle genomes in ferns (**Table 3**). In addition, based on Chang et al. (2009), both plastome and mitogenome are likely maternally inherited in *Dryopteris* ferns (including *Acrorumohra*; Dryopteridaceae, Polypodiineae, Polypodiales). Given that apomictic fern gametophytes can only produce sperm cells (Gastony and Windham, 1989), *Dryopteris diffracta*, which produces 32-spored sporangia and is indicated as an apomictic species, should be the paternal parent of *D. × subreflexipinna*, while *D. hasseltii* should be the maternal parent. In Chang et al. (2009), they found all individuals of *D. × subreflexipinna* had identical ptDNA and mtDNA sequences to those of *D. hasseltii*. Taken together, so far, there is no known exception of maternal inheritance of the organelle genome in ferns as well as in seed-free land plants (**Table 3**; Zhang and Sodmergen, 2010). For land plant organelle genomes, biparental inheritance and RNA-editing are both suggested as important mechanisms to rescue deleterious DNA mutations or effects due to nucleocytoplasmic incompatibility (Zhang and Sodmergen, 2010; Castandet and Araya, 2012). It is worthwhile to further examine whether biparental inheritance possibly evolved as an alternative rescue mechanism in the seed-free plants known with no or relatively infrequent RNA-editing, such as Osmundales and Marattiales ferns (Knie et al., 2016). The results from these plants will better shed light on whether paternal transmission or biparental inheritance is restricted in seed plants.

## Author Contributions

L-YK designed and carried out the experiments, analyzed the data, and prepared the manuscript. T-YT assisted on the experiments. F-WL and H-JS assisted on genome assembling, and SNP identification. W-LC, Y-MH, and C-NW discussed the experimental design and results, and provided the facilities to accomplish this work. All authors commented and revised the manuscript.

## Conflict of Interest Statement

The authors declare that the research was conducted in the absence of any commercial or financial relationships that could be construed as a potential conflict of interest. The reviewers WN and YS and handling Editor declared their shared affiliation.
