# Supplementary material for: Organelle Genome Inheritance in Deparia Ferns (Athyriaceae, Aspleniineae, Polypodiales)
Source: Front Plant Sci. 2018 Apr 13;9:486. doi: 10.3389/fpls.2018.00486 (PMC5932399; doi:10.3389/fpls.2018.00486)
Supplement: Supplementary file 1 [file Data_Sheet_1.DOCX]

**FIGURE S1|** Alignment of the partial *ndh*F gene, the ptDNA marker applied in this study. The alignment includes two parents (A and B) of *Deparia lancea* (GenBank no.: MG972639-40) and *Woodwardia unigemmata* as a reference (GenBank no.: KT599101 position 107909-107627). The gray block indicates the recognition site of the AciI enzyme.





**FIGURE S2|** Alignment of the partial *nad*9 gene, the mtDNA marker applied in this study. The alignment includes two parents (A and B) of *Deparia lancea* (GenBank no.: MG972637-38) and *Asplenium nidus* as a reference (GenBank no.: AM600641 position 14288-14854). The gray block indicates the nucleotide site distinguishing the two parents





**FIGURE S3|** Alignment of the partial *IBR3* gene, the nDNA marker applied in this study. The alignment includes two parents (A and B) of *Deparia lancea* (GenBank no.: MG972633-36) and *Deparia × lobatocrenata* (cDNA sequence, GenBank no.: KR826619 position 1694-2048) as the reference. The positions of non-coding regions, of the 12^th^, 13^th^, and 14^th^ introns, are according to the annotation of *Athyrium filix-femina IBR3* (GenBank no.: KF553740). The gray block indicates the nucleotide site distinguishing the two parents.
